# Editorial

**Published:** 1861-12

**Authors:** 


### Editorial.

"The Chicago Medical Examiner," for 1862.—With the present number we issue a full index to the volume of 1861. The next issue will commence a new volume; and notwithstanding the war, we trust that all our old subscribers will continue, and many new names will be added to the list. Indeed, the number of remittances already received from new subscribers to commence with the volume for 1862, is most gratifying. There will be no change in the editorial or other management of the Examiner for the coming year. In addition to ordinary communications, we shall continue to publish faithful reports of the doings of our local medical societies and colleges; and, also, many of the clinics in the hospitals and dispensaries.

Whatever we deem calculated to advance the education of the profession, or the science and art of medicine, we shall commend and sustain, without regard to the source from which it comes. We shall not imitate some of our neighbors by filling our columns with personal scandal, and cowardly insinuations. For we believe the almanac which says, "The man who tries to build himself up by pulling others down, is like one who sits himself on a wheelbarrow, and tries to wheel himself to glory."

The unhappy state of the country has caused many of our exchanges to stop publishing; but the Examiner will not stop on account of war and rebellion at home. If, however, England, or any other powerful European nation should be ungenerous enough to take advantage of our domestic strife, to commence an unjust war upon our country, we might be constrained, not only to lay down the editorial pen, but our whole *professional* armor, also; and to shoulder a knapsack and rifle to aid in defending the honor and integrity of our country until the last foe was driven from the field.

VALUABLE DONATION.—The Library of the Medical Department of Lind University has been enriched by a copy of *Rosenmueller's Abildungen der Anatomie*,—The donation of Dr. J. J. LESCHER, of Mt. Carmel, Ill.

We give place here, at the request of the Faculty, to the following extract from the minutes of the proceedings relative to the receipt thereof, as also to the interesting letter of Dr. LESCHER:

"A letter was received from Dr. J. J. Lescher, of Mt. Carmel, donating to the school a valuable work on Surgical Anatomy, with plates, and text in German and Latin. On motion of Prof. Davis, it was—

*Resolved*, That the Faculty of the Medical Department of Lind University tender their cordial thanks to Dr. J. J. Lescher, of Mt. Carmel, Ill., for his very valuable donation to the College Library.

It was further resolved that the Secretary embody the resolution in a note to the Corresponding Secretary, for transmission to Dr. Lescher."

The following is Dr. LESCHER's letter accompanying the volumes:

MOUNT CARMEL, ILL.,<br>*Nov.* 13*th*, 1861.

Prof. WM. H. BYFORD, M. D., Chicago—

*My Dear Sir*:—I take the liberty to request you to present in my name, to the Medical Faculty of Lind University, for the use and behoof of whomsoever they will, the accompanying copy of *Rosenmueller's Abildungen der Anatomie*, together with the *Text* in Latin and German—descriptive of the *Illustrations*, including the Surgical Anatomy of the same, the *Chirurgicsh Anatomische Abildungen.*

Intrinsically valuable as are these works of art, both by reason of their rarity and the beauty and truthfulness of the delineations, they possess yet greater worth in my sight, inasmuch as they descended to me from my revered and beloved father, of whose large library, accumulated during a half century at great labor and expense, they formed a part.

Believing, however, that they may become more useful in being placed under the control of the worthy teachers of a young and vigorous institution devoted to the spread of "the heaven-descended art," than to remain in a private library, I am induced to make the above named disposition of them. In this connection I may be allowed to speak of my father:—

Born in 1783, a descendant of Swiss parents,—his father born on the North Atlantic ocean,—my father began collecting a library as early as 1800. At the date of his death, in 1854, it numbered 994 volumes—costing from $1.00 to $87.50 each. Possessed of no means in early life, with but six months "schooling," (in German) to gratify his taste for reading, he was fortunate in forming the acquaintance of learned men of Lancaster, Penn., chief amongst whom was the pioneer of the German Lutheran church, in America, the Rev. Dr. Henry Mühlenburg, the founder of a family since noted in theology and legislation.

Possessed of a large and rare collection of books, Macænas-like, he encouraged my father, giving him free and generous access to his library. Residing some twenty miles in the country, in the pursuit of a miller's boy, he would frequently walk the distance to borrow a book of the Rev. Dr., for perusal at night, after the toils of the day, and as he could raise money, he would purchase of him an occasional volume.

Of these evidences of his early tastes and studies, I now possess several samples, which I prize highly, chiefly, because of their associations.

One of the rarest works, now in my library, which he purchased of a monk, is in 13 vol. folio, containing 23,582 pages, double column, German text, with Latin notes, richly bound, and beautifully printed, entitled, "*Historisch-Politisch Geographischer Atlas der Grantzen Wett; order grosses and vollstandisches Geographisch—und Critisches Lexicon. Von Mr. Brouzen La Martintere.*" *Leipzig*, 1744.

With my best wishes for the Faculty respectively, and the University, which is their pride,

I am very truly, your friend,

J. J. Lescher.

Army Sanitary Commission.—One cannot resist the conclusion, from the perusal of the various publications of this body, that a great laxity, even to criminality, exists in some of the regular machinery of the War Department, that thus necessitates the organization and maintenance of a charitable institution of such magnitude as this,—for to this eleemosynary complexion has it come at last.

Organized originally, as we were led to believe, mainly as an advisory body to the Medical Bureau, it has grown in importance and value until it now actually rivals that institution,—not alone in the furnishing of hospital supplies and stores, but in the organization and operation of the hospitals themselves; and,—we regret that it should be so,—the field for these guerrilla Samaritans is so ample, that, as yet, conflicts of authority, between them and the army medical officers proper, are matters of rare occurrence. That such a fact should be, is a stigma upon the Medical Bureau and its officers; that two-thirds,—certainly one-half of the sick and wounded should be dependant upon the chance donations of the public, already taxed to support an organization, ostensibly for this purpose, is a crying shame upon the Department of which that Bureau is a creation. And the evil ramifies down, from the chief of that Department, to the private in the ranks, who, knowingly unfit for duty, either actively or passively, burdens himself and his infirmities upon the Government. We know of officers, who, to obtain their commissions, have had mustered into their commands, to attain the necessary number, men totally unfit for service, with running sores, with herniæ, with carious tibiæ, etc.; of others, who have thrown difficulties in the way of their sick and wounded being discharged, for similar reasons. One man, we saw, only a short time since, who has helped fill up *three* companies, obtaining his discharge each time, on account of a large open ulcer over a carious tibia. It is not necessary to say that the fellow found his account in this trick, through the various captains, or that it was done through their cognizance, the matter being one of seller and purchaser,—and in which the ulcer and the Government were alike sold.

Would such a trick be feasible,—would such a state of affairs obtain,—would we have "20,000 unfit for duty" in a single division of the army, if mustering and inspecting officers did their duty? And if not, and other officers from the inspector up to Surgeon-General FINLEY were competent and faithful, would there be any need of such a stupendous Charity Society as the *U. S. Sanitary Commission* has degenerated into?

We presume there is no necessity for us to explain our motives in these remarks. They are made in no captious spirit; nor will we yield to any in lauding the self-sacrifice and public zeal which prompts the members of the *Commission;* still less do we underrate the value of the good they do. What we regret and object to, we have clearly stated:—the *necessity* for such self-sacrifice and exertion, and the existence of such suffering, destitution and inefficiency as their reports show.

Incidentally, we may mention that the Chicago Branch of the *Commission* has furnished all the hospitals at Cairo with clothing, bedding, food, and miscellaneous articles, and has in store at that place, 120 boxes more, in reserve for an emergency. It has also supplied the hospitals at Rolla, Sedalia, Tipton, Otterville and Bird's Point, Mo., Paducah, Ky., Mound City, Ill., and Ft. Leavenworth, Kansas. Some fifteen large boxes of these goods are daily received, from all parts of the State, and from twelve to fifteen dispatched to the various points above mentioned. Through its instrumentality much good has been done in the various camps, in enforcing sanitary and police regulations, in seeing to the rations and modes of cooking, etc., aside from the more strictly medical province, in which much has been done in the way of weeding out irregular practitioners, instituting order, cleanliness and discipline, and otherwise ameliorating the condition alike of the sick and well.

EXTRACTS FROM CORRESPONDENCE.—Dr. S. W. WALLACE, writing us under date Oct. 28, 1861, mentions that he had recently kept a lady patient,—whose hand had been so severely crushed as to demand amputation at the wrist,—under chloroform for two hours, and without any bad result.

—A New York correspondent sends us a sketch of the introductory lecture to the tenth annual session of the New York Ophthalmic School, delivered at the hospital, No. 63 Third avenue, by Dr. M. STEPHENSON, before a large number of students and professional friends. The address was one of much interest, and we are glad to know that the school is doing well.

"With over a thousand patients per annum at the Institution, great facilities are always afforded for clinical instruction.

The attending surgeons are Dr. M. Stephenson, Dr. J. P. Garrish, and Dr. M. P. Stephenson.

The hospital is always open to professional brethren, especially those who may visit the city from a distance."

G. G. S.

—The following we take from a letter from C. DUMREICHER, M. D., formerly of this city, but at present Assistant-Surgeon at the General Hospital, Wheeling, Va. Dr. DUMREICHER served through the campaign with Gen. Rosencranz, in Western Virginia, and has been in the service from an early date in the war. He says:—"The time that I have lived in the field has been extremely interesting. I have seen and practiced more surgery and medicine in this short time, than I would have been able to do, in civil practice, in a much longer period, and I have seen some very interesting cases. At the battle at Carnifax Ferry, the hospital of our wounded was under my charge. One man was shot through the chin. The ball fractured the lower jaw terribly; passed along the lower border of it, and stopped immediately above the external carotid. After a very careful and delicate dissection, I extracted the ball. The loss of blood from the wound was frightful, and I thought the man would die; but he recovered, and two weeks afterwards, I sent him to the General Hospital in Cincinnati. Another man was shot through the upper lobe of the right lung, and got well. I did not lose a single wounded man after that battle, except the amputations, and these were nearly dead before the operation. At Gauley

Bridge, a very singular case was under my treatment. A man, sick with typhoid fever, was sent from the camp, about six miles above my hospital, to me for treatment. On the road, he was shot from across the river. The ball entered his body at the lower border of the right scapula, glanced off, and took its direction upwards, through the neck, went under the lower jaw, across the mouth diagonally, fractured the left superior maxillary, and lachrymal bones, and was cut out by me, immediately below his left eye. It was a heavy Minié rifle ball. There was some loss of blood from the mouth, but not much otherwise. The muscles of the tongue were somewhat cut, but not very badly. No important artery or nerve injured. A very strange case. The man got well. Another soldier's tibia was injured by a shot, and fractured by a subsequent fall. Several surgeons decided to amputate, and sent to me for it. The fibula was intact, no artery or nerves cut, and I made up my mind to save his limb, and succeeded.

* * * * Brig. Surg. Clendennin, whom you know, is surgeon of this hospital. At the present time, I am prescribing for between 70 and 80 patients, every day.

Army Medical Society at Cairo.—We learn from the St. Louis *Republican*, that an association, called the Army Medical Society, has been formed at Cairo, composed of the surgeons and assistant surgeons connected with the various regiments and hospitals at Cairo, Bird's Point, and Mound City; its object being the improvement and diffusion of medical science among the members. The preliminary meeting was held at the office of Dr. Taggart, the medical purveyor at that post, and was temporarily organized by Surgeon Stahl being called to the Chair, and Brigade-Surgeon Burke appointed Secretary. Brigade-Surgeon Brinton explained the object of the meeting as being one in which all the surgeons, now engaged in the army, should feel a deep interest during the war. Surgeon Bringhurst moved that a committee be appointed to report a list of officers for the government of the Association. The following gentlemen were elected: Surgeon Stearns, President; Vice-Presidents, Surgeons Davis and

Bowman; Secretary, Surgeon Taggart. They will hold a meeting once a week, and two papers will be read at each, for discussion; and every subject connected with army surgery will be discussed.

WORK ON "NEW REMEDIES."—In connection with Prof. PERCY, an extract from whose pen we publish in the present number, Dr. ELSBERG, of the *American Med. Monthly*, is preparing a work on *New Remedies*. Dr. E. says: "It is based on the celebrated Essay of Dr. GUIBERT, to which the *Société des Sciences Médicales et Naturales de Bruxelles* awarded its full prize, and the erudite elaboration thereof by Dr. RICHARD HAGEN, now in course of publication at Leipzig, Germany. Our work will embrace all valuable medicinal agents introduced into the treatment of disease since the year 1830, up to the present day, detailing their history, description, action and uses, and giving the most approved Formulæ of Preparation, Preservation, and Administration. In Formulæ it will be particularly full, for the use of both physicians and pharmacists. Novelty not being deemed a sufficient passport for admission into confidence, unless sustained by merit, and with the only object—*to be useful*—and the only means—*Labor to approach the Truth*—constantly before us, we are determined that no really useful remedy, introduced during the last thirty years, shall be slighted, while no undue prominence shall be given to undeserving articles. Nor will uncertainty or ignorance at any point that the actual advance of science has not reached, be sought to be concealed by illusory hypothesis or ill-founded statements. Any heretofore unpublished information calculated to add to the practical utility of the work, that may be in the possession of any of our readers, will be gratefully received, carefully considered, and, if used, appropriately acknowledged."

OBITUARY.—Among the recent deaths of eminent men in our profession, is M. SCRIVE, Chief Inspector of the French Army, and Medical and Surgical Historian of the Crimean Campaign. He died at the hospital *Val de Grace*, Paris, ætat 46.

Also Sir JOHN FORBES, M. D., near Reading, England, well-known, aside from his other writings, by his volume on *Nature and Art in Disease*, and the discussion it gave rise to.

New Applications of the Per-Chloride of Iron.—Dr. Rodet, of Lyons, claims that a solution of per-chloride of iron will destroy the virus of hydrophobia, and recommends it as a specific after the bite.

M. Wahu, principal physician of the Military Hospital, at Nice, reports the successful treatment of an *ongle encarné*, or ingrowing toe-nail, which had partially disabled him for a long time, by the use of the salt, after vainly trying the Vienna paste and alum. The powder was obtained and insinuated as deeply as possible between the free edge of the nail and the ulcer; fifteen minutes after, the Dr. could bear his entire weight on the foot, a thing he had been unable to do before for many months. Three weeks after, by means of a bath, he removed the hardened layer of tanned skin, and found a new tissue beneath which perfectly resisted the pressure of the nail.

Other continental physicians testify to the efficacy of the salt, in various cutaneous affections. M. Bouron des Clayes, of Créteil, reports two cases,—the first of an old man, suffering from eczema and an intolerable itching in the perineal region, unallayed by starch poultices, tar-ointments, etc. The liquid per-chloride was lightly applied twice, at an interval of twenty-four hours, each time coated over with collodion. The third day the cure was completed. The second case was that of a strong man, forty-seven years old, suffering from itch, and also, an eruption of *lichen agrius*, of long standing, on the fore-arm. An ointment of the per-chloride, bicarbonate of soda and hog's-lard was applied, and in a few days the itch had completely disappeared, the eruption was subsiding, and a cure was rapidly effected.

Physician's Hand-Book, etc.—From W. A. Townsend, 39 Walker street, New York, we have received the *Physician's Hand-Book of Practice*, for 1862. This is the well-known little volume of Dr. Elmer, and contains, in addition to the usual diary for medical practice, which is very full and complete, an amount of condensed information which the young practitioner will find very useful.

Townsend also re-publishes *Braithwaite's Retrospect*, of which we need not, here, do more than speak,—preferring, as heretofore to notice it, *in extenso*, on receipt of the number.

Mortality in Chicago.—The following table shows the mortality in each division for each month of the years, 1860–61. The total mortality for the past three years has been 1,862 for '59, 2,056 for '60, and 2,089 for '61.

| | 1860. | | | 1861. | | |
|---|---|---|---|---|---|---|
| | North Div. | West Div. | South Div. | North Div. | West Div. | South Div. |
| January | 25 | 48 | 42 | 63 | 59 | 53 |
| February | 31 | 61 | 36 | 36 | 53 | 46 |
| March | 38 | 74 | 68 | 43 | 73 | 56 |
| April | 44 | 48 | 39 | 36 | 46 | 44 |
| May | 32 | 40 | 30 | 43 | 44 | 47 |
| June | 41 | 66 | 48 | 36 | 45 | 50 |
| July | 95 | 116 | 77 | 67 | 85 | 87 |
| August | 86 | 122 | 100 | 44 | 126 | 92 |
| September | 43 | 69 | 60 | 47 | 98 | 78 |
| October | 34 | 65 | 50 | 28 | 88 | 50 |
| November | 58 | 52 | 60 | 39 | 60 | 64 |
| December | 46 | 63 | 49 | 40 | 83 | 72 |
| Totals | 573 | 824 | 659 | 522 | 810 | 739 |

Mortality among Medical Journals.—Since our last issue, the following periodicals have suspended publication: *North American Medico-Chirurgical Review*, *Berkshire Medical Journal*, *Cleveland Medical Gazette*, and *St. Louis Medical and Surgical Journal.* There are now just about one-third of the medical publications issued, that commenced the year.

The spasmodic character of whooping-cough is said to be thoroughly controlled by the exhibition of an infusion of common clover-hay, after the following formula:

℞ Trifolii in fœno, - - - ℥ij.

Aq. bullient, - - - - Oj.

Macerate for four hours and strain. Dose, a tablespoonful, three times a day, to a child five years old. The hay should be sweet and leafy; and this infusion is to be used in conjunction with other remedies, according to the indications.

Personal.—We understand that Dr. J. V. Z. Blaney, Medical Purveyor at this post, has been summoned to Ft. Leavenworth by Gen. Hunter, to assume his duties there as Brigade-Surgeon.

Cure for Whooping Cough.—Dr. B. Woodward, of Galesburg, Ill., uses a new formula with which he states to have succeeded beyond his most sanguine hopes. Take best roasted Java coffee, four ounces; boiling water, eight fluid ounces; displace and add loaf sugar q. s. to make a syrup. When nearly cold, add tincture of veratrum viride, two fluid drams; fluid extract of conium, four fluid drams. Give from one-half to one teaspoonful every four or five hours, till the cough is well. This treatment will effect a cure in from eight to twelve days.—*Med. and Surg. Reporter.*

Dupuytren on Spurious Pregnancy.—Prof. Simpson, in lecturing on spurious pregnancy, tells the following good story of Dupuytren. "Such cases are, happily, extremely rare; but you do meet with them occasionally, and not in patients within the walls of a lunatic asylum only. It is told that a lady once came to Dupuytren, to ask what was to be done in her case, as she had now been in the family way for fourteen years—and the great Parisian surgeon gave it as his opinion, that as the boy must be tolerably well grown by that time, the best thing the lady could do was to swallow a tutor immediately, that his education might not be neglected."—*Med. News & Library.*

—— Professor Peaslee recently performed the operation of tapping, on a young lady, at Pittson, Pa., and removed *one hundred and forty-nine pounds and three ounces* (149 ℔s., 3 oz.) of dropsical fluid. It was weighed in Dr. Peaslee's presence, by Dr. Lawson and Mr. J. Loveland, of that place. The abdominal circumference of the patient before the operation, was *six feet and two inches*, (74 inches.)

This was the same patient, mentioned in the *Am. Med. Monthly*, June, 1861, from whom Dr. Peaslee removed *one hundred and thirty-five pounds* of fluid (135℔s) on the 29th of April last. The circumference then, was five feet and seven inches, (67 inches.)

Physical Training.—Among the Parliamentary papers recently issued, are two small volumes containing some information collected by Mr. Edwin Chadwick, during the recent education inquiry Mr. Chadwick shows in these papers that the present practice of long hours of teaching, is a wide cause of enervation and predisposition to disease, and induces, also, habits of listlessness and dawdling. The half-time system is found to give nearly, if not quite, as good education as the whole time; and common sense tells us that a boy who has acquired the same amount of knowledge in half the time of another boy, must have obtained proportionately superior habits of mental activity. It is the alertness, combined with the bodily aptitudes created by drill, that gives comparatively stunted boys of the town, a preference over the strong, robust lads from the coast. Good school-masters say, that about three hours a day are as long as a bright voluntary attention on the part of children can be secured, and that in that period they may be readily taught as much as they can receive; all beyond the profitable limit is waste.—*Med. Times and Gazette.*

Podophyllin as a Substitute for Mercury.—This article, the active principle of the *podophyllum peltatum*, *May apple*, or *mandrake*, is just now coming into notice among our transatlantic neighbors, as a substitute for mercury. The suggestion of its use came, however, it is said, from an American oculist, who, in 1854, when about to leave the English shores, placed his daughters under the care of a celebrated physician, and gave the latter a bottle of this remedy, which he requested should be prescribed instead of mercury, whenever a mercurial was required in their case. Following this suggestion, the physician has used it continually since, and thus speaks of its administration and effects. If given in quarter-grain doses, twice a day, combined with opium, to check its aperient action, and continued for a few days, profuse salivation will occur, with, however [no?] fetor of breath, or ulceration of gums. This, however, rapidly subsides on discontinuing the medicine. It is very slow in its action, often ten or twelve hours, but in the following combination induces one or two copious stools, attended with the sensation of the bowels having been thoroughly emptied, and without tenesmus:—℞. Podophyllin, gr. j.; pulv. rhei, gr. ix.; pulv. capsici, gr. ij. M. Ft. pil. iij. S. one or two. This, for an ordinary aperient. Its action on the liver, if given in small doses, may be as much relied on as mercury, while the effects upon the system generally are, by far, less injurious.—*Am. Med. Monthly.*

Gun-shot Wounds produced during the loading of Artillery.—Dr. Cortese relates (*Omodei Annali Univ. di Med.*) five cases, and gives the following summary of his observations. No other blow of a projectile imparts so great an amount of commotion to the entire limb, and the surgeon is therefore compelled to direct his attention to the whole extremity, whatever amount of lesion may be manifest in the hand. A neglect in this regard may lead to gangrene gaining possession of a large portion of the limb, or to a generalized suppuration, while a diminished power of re-action in the injured parts may give rise to purulent infection, or render amputation useless. When the hand is severely torn, its disarticulation and even the amputation of the forearm is insufficient to secure recovery, because the tissues are more or less destroyed in their intimate structure in consequence of concussion. In such cases, the arm should be amputated. The sooner amputation is performed, the greater is the probability of a favorable result. The rapid and very extensive tumefaction of the limb constitutes a sufficiently certain criterion of the severity of the derangements which are propagated along its whole extent. When no fractures are detected in the diaphysis of the bone, some lesion in the ulnar articulation must be suspected. When the lesion does not seem severe enough to call at once for amputation, we must be prepared for secondary occurrences which will unfit the limb for its functions. Still, conservative treatment should in such cases be attempted.—*Boston Med. & Sur. Journal* from *B. & F. Med.-Chir.*

Periosteal Reproduction of Bone.—It is claimed that Boyer anticipated Flourens, Ollier, and others in the discovery of the reproduction of bone by the periosteum. He says that in Boyer's work on practical surgery, "all the circumstances of the reproduction of bone by the periosteum are carefully enumerated, according to the origin, extent, or depth of the mischief," and presents the following among other quotations, therefrom, in proof of his statements: "When the periosteum of both surfaces of a bone has been spared, it inflames, the vascular net-work becomes more apparent, the thickness of the membrane increases, it parts from the modified structures, and the interval is immediately filled up by a gelatinous, or more properly an albuminous substance, at first semi-fluid and jelly-like, but subsequently more consistent and adhesive to the periosteum only. This albuminous layer becomes more compact and opaque; red patches are soon discernible, and it blends with the enveloping membrane from which it can no longer be distinguished. In this mass, streaks and layers of bony deposit, at first sparse and disseminated, are, after a time, visible; they increase in number and close with each other; the thickness and density of the mass daily become greater, and at the same time it is deflected and swerves from the mortified bone; for a long time it can be readily cut through, and the surface of the incision presents a cellular mixture of solid and apparently fleshy aspect; the osseous structure at last shows itself unmistakeably; *a new bone has been formed*, and the surface corresponding to the mortified fragment remains covered with a thin layer of soft texture, which replaces the internal periosteum.

Re-organization of the Medical Department of the Army.—Senator Wilson, of Massachusetts, has introduced a bill into the Senate, which looks to the attainment of the following ends :—

1. The introduction of an efficient corps of medical officers, especially devoted to sanitary inspection and hygienic administration.
2. The selection and appointment of the Chief of the Medical Bureau—the Director-General—as well as all the Sanitary Corps, solely with reference to *fitness.*
3. The establishment of a rule for the honorable retirement of every medical officer, at a specified age of presumed inability.
4. The recognition of, at least, one degree of higher rank in the medical staff.
5. A better definition of the various ranks in the medical staff, and a systematic assignment of general departments of labor to each rank or class of surgeons.
6. An increase of the class of Medical Cadets, and a proper recognition of their status and privileges in the medical staff of the army.

Meanwhile, Congress is quarrelling over the admission of homœopaths into the army hospitals, and two of the highest regular Medical officers have been rating each other soundly, while a volunteer association is inspecting hospitals and camps, feeding, attending and clothing the sick, and in general, performing the duties appertaining to the regular Medical Department.

Treatment of Child-Bed Fever.—In the epidemic of child-bed fever which occurred some time ago, in the obstetrical clinique of Professor Von Ritgen, the following plan of treatment was adopted, with exceedingly beneficial results, as even cases of the utmost severity were cured under its influence. At first $\frac{1}{8}$th of a grain of morphia was given, and this dose repeated two, three, or even four times a day, according to the violence of the abdominal pain. An hour after the dose of morphia, a mixture of camphor was admistered :

℞—Camphor, - - - - ℈ss.

Gummi mimos., - - - ℥j.

Aq. Chamomill., - - - ℥iij.

Liq. Ammon. acet., sacch. albi, aa., ℥j.

An hour after this the patient took one grain of quinine; then another dose of morphia, and so on, until the symptoms decreased, which was the case with all patients hitherto treated in this manner.—*Med. Times & Gaz.*

Regeneration of Osseous Tissue.—We copy the following from the *San Francisco Med. Press*:—

"The following review of the researches of M. Ollier in this department of Physiology, which is daily becoming more and more interesting, from its important bearing in reference to Operative Surgery, we translate from *Canstatt's Jahresbericht*, 1st vol., 1861:

The part which the periosteum performs in the regeneration of osseous tissue, Ollier attempts to show, by a series of interesting experiments. In order to resolve the question, whether portions of bone could be reproduced outside of the normal limits and regions of ossification, as well as by the agency of blood-vessels, to which such function does not normally belong, Ollier attempted and effected transplantation of portions of the periosteum, taken from the living bone. His experiments were performed upon rabbits, in which he detached portions of periosteum in such a manner, that a part of them was left with its original attachments, while the other was separated and drawn out through the muscles. In another series of experiments, these flaps of periosteum were detached from the bone after a few days; finally, in a third set of experiments, the flaps were wholly detached from the bones, and were transplanted upon adjacent and also on remote portions of the body. In all cases, in connection with the periosteal flap, there was found a new layer of bone, of different degrees of strength, corresponding to the dimensions of the flap: this new bone consisted of a compact cortical structure and of medullary cells, the latter finally uniting in a large medullary cavity. The newly formed bone, independent of a slight irregularity of the Haversian canals, exhibited the same structure, when examined microscopically, as ordinary bone: it had a red marrow, rich in vessels, and similar to that of the fœtus. The formation of the new bone does not proceed from the fibrous portion of the periosteum, but from a thin layer of blastema, which covers the inner superfices of the periosteum, and contains free cells, nucleated cells, and an amorphous, granulated material. Where the blastema was rubbed off from the inner surface of the periosteum, there occurred no deposit of bone; when the fine fragments of this blastema were strown upon other tissues, they produced bone cells, without the aid of the fibrous portion of the periosteum.

The course of the formation of bone is not, throughout, constant and similar; during the first few days, the flaps of periosteum, as well as the surrounding tissues, become swollen, on account of the infiltration of lymph; soon afterward, there appear, on the inner surface of the periosteum, masses of matter deposited, distinguishable on account of its consistence: the deposition of calcareous matter commences about the seventh or eighth day. The mass, impregnated with calcareous matter, is sometimes fibrous in structure; at other times it is of a cartilaginous nature, the cellular spaces of which usually contain a single simple cell, or else fine granules. The bone, when stripped from its periosteum, is soon again invested with a thin, delicate layer of matter, which soon unites with the wounded edges of the old periosteum: in eleven or twelve days afterward, the formation of vessels appears, in the new layer of matter, the vessels arising from either the edges of the wounded periosteum, or from the bone itself: along with the vessels, there are also produced connective tissue and elastic fibrous matter. In six or seven weeks, this newly formed periosteum acquires the power, similar to the original one, of generating masses of bone in any part of the body; still, it should be stated, that this reproductive attribute is not possessed, to an equal extent, by all portions of the periosteum. For example, after transplantation of the pericranium, there appeared but a very diminutive osseous granulation; on the other hand, the dura mater, (as much of it as is in connection with the cranium,) when transplanted beneath the skin of the sides and other regions of the body, in 30 or 40 days afterwards, produced fragments of bone, of from 2 to 6 millimetres of thickness. (A millimetre equals a half line.) The flaps of the periosteum retained the power of reproducing bone, when they had been detached 10, 30, 60 and even 99 minutes after the heart of the animal from which they were taken had ceased to beat. An entire humerus was taken from a rabbit that had been killed, and was inserted beneath the skin of another living rabbit; the bone not only grew fast in its new place, but increased in length and breadth, the growth in the latter direction being greater. When the bone was taken from an animal of another species, the operation failed; the transplanted bone either causing an abscess, or, after a time, disappeared by absorption.

Belladonna Shortening Labor.—Dr. B. F. Barker gives a table of 147 cases of labor, in all of which belladonna had been given for the purpose of dilating the os externum by comparatively painless contractions. The extract was given in one-quarter-grain doses, two or three times a day, commencing about two weeks before the end of gestation. Plethoric patients took tartar emetic in combination with belladonna—three grains of the former, eight of the latter, in two ounces of the syrup of orange peel, one ounce of the tincture of orange peel, and one ounce of water; a teaspoonful three times a day. With some the following formula was used: compound tincture of cinchona, three ounces; syrup, one ounce; extract of belladonna, eight grains. Other combinations were prescribed to fill special indications.

A very great difference appeared in the susceptibility of patients to the influence of the agent, and also a great difference in the purity and strength of the article. One would seem to have double the potency of another, without any corresponding difference in the appearance, color or odor. In some cases the dose had to be diminished, but in most instances it could be gradually doubled, or even tripled. Dryness of the throat, slight uneasiness or giddiness in the head, dimness of the vision, are indications to diminish the dose. Not one of the children was still-born, and in none of the cases was there post-partum hæmorrhage or retention of the placenta. In one the function of lactation was entirely absent; in two others the mammary secretion did not appear until the fifth day.—*Amer. Med. Jour.*

Puerperal Convulsions.—Dr. T. J. Thomas, in one of his lectures on the complications of labor, delivered in the University Medical College, New York, thinks the term puerperal should convey the idea of eclampsic seizures, of epileptiform character. Poisoning of the blood, usually uræmia, is, in a vast majority of cases, the great cause of the seizures. As a rare exception to this rule, centric or eccentric irritation may act as a cause. The avenues by which death may approach, are apoplexy, asphyxia, serous effusion, coma, exhaustion and paralysis of the heart. Consequently, the primary indications of treatment may be enumerated as follows:

1. Check the convulsive action at once and thus prevent death by asphyxia, the cerebral conditions resulting from congestion, and failure of the heart to perform its function.

2. Diminish vascular turgescence, and excessive action, and thus remove the great liability to apoplexy and coma.

3. Evacuate the uterus, if possible, because experience has proved that, in the majority of cases, the seizures will then cease, and because we thus remove pressure from the kidneys.

4. Eliminate or neutralize the poison accumulated in the blood.

Anæsthesia by chloroform, resorted to early and fearlessly, is by far the best means to control the convulsions, but its influence must be kept up steadily and unintermittingly, even for twenty-four or forty-eight hours, if necessary, and in such manner as to effect the object in view, if it can be effected by this means. When anæsthesia fails to control the convulsions, blood-letting should be taken into consideration. It may be necessary, but its indiscriminate employment is very injurious. In regard to the third indication, labor, if actually commenced, should be encouraged and hastened, so soon as the convulsions are at all controlled. Should the woman not be at term, an attempt must be made to manage the case without the induction of labor; but the latter should be accomplished when other means fail to prevent the return of the seizures. For this purpose, pass a sponge-tent into the os uteri, use the warm douche freely against this and the encircling fibres of the os, and employ the colpeurynter or a bladder placed in the vagina and filled with water. Should the os be dilated, stimulate the fibres of the uterus by placing a gum-elastic catheter between the membranes and the uterine body, or deliver by version or the forceps. The kidneys being crippled in their functions, it is finally advisable to act freely on the mucous membrane of the alimentary canal by active cathartics—salines, if the patient can swallow, otherwise croton oil. The skin should be made to act by the hot-air or vapor bath. In addition, dilute citric or benzoic acid should be freely given, with the hope of forming in the blood citrate of benzoate of ammonia. The former may be given in the form of lemonade.

As cases complicated with œdema are much more favorable than those without it, some benefit may be derived from the artificial production of œdema, by ligatures applied around the arms and legs tight enough to interfere with venous return, but not to obstruct arterial flow. Sometimes in the convulsions occurring after delivery, opium, in full dose, is highly useful, but its use requires great caution.—*Am. Med. Monthly.*

Tartar Emetic in Asthma.—In three cases of long standing, Dr. E. B. Forsee obtained permanent relief by nauseating doses of tarter emetic with morphia: one-fourth or one-eighth of a grain of the one, one-eighth of a grain of the other, repeated every twenty, thirty or sixty minutes. When the spasmodic action abates, the patients are advised to take small doses of antimonial wine for some days.—*St. Jo. Med. and Surg. Journal.*

